# Physiological and Proteomic Changes in *Camellia semiserrata* in Response to Aluminum Stress

**DOI:** 10.3390/genes15010055

**Published:** 2023-12-29

**Authors:** Junsen Cheng, Tong Li, Shanglin Wei, Wei Jiang, Jingxuan Li, Yi Wang, Yongquan Li

**Affiliations:** 1 College of Horticulture and Landscape Architecture, Zhongkai University of Agriculture and Engineering, Guangzhou 510225, China; a416531623@163.com (J.C.); annlee6133@163.com (T.L.); weishanglin2000@163.com (S.W.); 15329524602@163.com (W.J.); 15656347778@163.com (J.L.); 2 Scarce and Quality Economic Forest Engineering Technology Research Center, Guangzhou 510225, China

**Keywords:** *C. semiserrata*, aluminum stress, proteomics, differentially expressed proteins, physiological response

## Abstract

*Camellia semiserrata* is an important woody edible oil tree species in southern China that is characterized by large fruits and seed kernels with high oil contents. Increasing soil acidification due to increased use of fossil fuels, misuse of acidic fertilizers, and irrational farming practices has led to leaching of aluminum (Al) in the form of free Al^3+^, Al(OH)_2_^+^, and Al(OH)^2+^, which inhibits the growth and development of *C. semiserrata* in South China. To investigate the mechanism underlying *C. semiserrata* responses to Al stress, we determined the changes in photosynthetic parameters, antioxidant enzyme activities, and osmoregulatory substance contents of *C. semiserrata* leaves under different concentrations of Al stress treatments (0, 1, 2, 3, and 4 mmol/L Alcl_3_) using a combination of physiological and proteomics approaches. In addition, we identified the differentially expressed proteins (DEPs) under 0 (CK or GNR0), 2 mmol/L (GNR2), and 4 mmol/L (GNR4) Al stress using a 4D-label-free technique. With increasing stress concentration, the photosynthetic indexes of *C. semiserrata* leaves, peroxidase (POD), superoxide dismutase (SOD), catalase (CAT), soluble protein (SP), and soluble sugar (SS) showed an overall trend of increasing and then decreasing, and proline (Pro) and malondialdehyde (MDA) contents tended to continuously increase overall. Compared with the control group, we identified 124 and 192 DEPs in GNR2 and GNR4, respectively, which were mainly involved in metabolic processes such as photosynthesis, flavonoid metabolism, oxidative stress response, energy and carbohydrate metabolism, and signal transduction. At 2 mmol/L Al stress, carbon metabolism, amino sugar and nucleotide sugar metabolism, and flavonoid metabolism-related proteins were significantly changed, and when the stress was increased to 4 mmol/L Al, the cells accumulated reactive oxygen species (ROS) at a rate exceeding the antioxidant system scavenging capacity. To deal with this change, *C. semiserrata* leaves enhanced their glutathione metabolism, drug metabolism-cytochrome P450, metabolism of xenobiotics by cytochrome P450, and other metabolic processes to counteract peroxidative damage to the cytoplasmic membrane caused by stress. In addition, we found that *C. semiserrata* resisted aluminum toxicity mainly by synthesizing anthocyanidins under 2 mmol/L stress, whereas proanthocyanidins were alleviated by the generation of proanthocyanidins under 4 mmol/L stress, which may be a special mechanism by which *C. semiserrata* responds to different concentrations of aluminum stress.

## 1. Introduction

Soil is among the necessary environmental materials used by trees for nutrients and survival. According to the United Nations Global Soil Pollution Assessment 2021, 33% of soils worldwide are currently experiencing moderate to high levels of degradation. Soil pollution is caused by a variety of factors, and soil pollution caused by acid deposition is particularly serious. If acid deposition exceeds the natural capacity of the soil to neutralize substances, some cations (e.g., Al^3+^) will be released from the clay structure and the organic mineral complex into the soil solution [1]. These cations will be absorbed and transported by some plants into their bodies, causing toxicity and seriously inhibiting plant growth and development [2]. According to statistics, approximately 2.5 billion hm^2^ of arable land and potentially arable land worldwide is acidic soil, accounting for approximately 50% of the total area of arable and potentially arable land [3]. In China, acidic soils are mainly distributed in red soil areas with high temperatures and rainfall in the south, covering 14 provinces (autonomous regions and municipalities directly under the central government) [4].

Apart from oxygen and silicon, aluminum is the most abundant element in the Earth’s crust, accounting for approximately 8% of the total elements in the Earth’s crust [5]. Aluminum is not needed for biological systems to function, and, simultaneously, there is no direct scientific evidence that organisms use aluminum in their biological processes, which remains an unsolved mystery. Normally, aluminum is found as insoluble silicates and aluminum oxides in neutral or moderately acidic soil conditions and exhibits no harmful effect on plant bodies [6]. However, due to atmospheric pollution and irrational agricultural practices, soil is eroded by acid rain or other factors, resulting in a pH value below 5.5. Compared to the content of metal cations (Mg^2+^, Ca^2+^, Na^+^, K^+^), the content of protons (H^+^) and strong acid anions (e.g., Cl^−^) is higher in acidic soils; as a result, acid deposition is far greater than the soil’s natural neutralizing capacity, and aluminum leaches in the soil solution. As a result of the leaching of aluminium from soil solutions, where some of the insoluble aluminium is dissolved as free Al^3+^, Al(OH)_2_^+^ and Al(OH)^2+^, free aluminum ions can be absorbed by the plant root system and cause the plant to show symptoms of Al toxicity [7], affecting its growth and normal metabolism [8,9].

As the problem of soil acidification has become increasingly serious, the yield and quality of agricultural crops and economic forests have decreased due to elevated concentrations of aluminum ions in the soil, progressively captivating the interest of researchers. Kopittke’s study showed that the root growth of soybean (*Glycine max* (Linn.) Merr.) was inhibited within 5 min at a concentration of 75 μmol/L Al [10]. In addition, Al stress affects the normal metabolic activities of plant roots and inhibits the growth and development of primary and lateral roots, causing primary roots to thicken and shorten; root tips to expand and become brown; and lateral roots and root hairs to reduce or even disappear [11]. As a result, the uptake of other essential nutrients by the plant roots is affected [12]. Al stress also induces an imbalance in ROS production in plants, causing the antioxidant enzyme system (e.g., superoxide dismutase (SOD), peroxidase (POD), catalase (CAT), ascorbate peroxidase (APX), etc.) to initiate a protective strategy to maintain cellular ROS homeostasis [13]. Several studies have shown that oxygen free radicals generated by excessive accumulation of ROS under Al stress contribute to lipid peroxidation, causing irreversible oxidative damage to cell membranes and ultimately leading to programmed cell death [14], a process that typically increases the synthesis of malondialdehyde (MDA) [15]. In addition to the role of antioxidant enzyme systems, intracellular osmoregulatory substances, such as soluble protein (SP), soluble sugar (SS), and proline (Pro), can trap, neutralize, and scavenge excess intracellular free radicals, thereby reducing the extent of oxidative stress [16,17]. The toxic effects of Al toxicity on the aboveground parts of plants should not be ignored. Excess aluminum ions inhibit the activities of key enzymes involved in photosynthesis in tea (*Camellia sinensis*) leaves and reduce the net photosynthetic rate (P*_n_*), intercellular CO_2_ concentration (C*_i_*), stomatal conductance (G*_s_*), and transpiration rate (T*_r_*) [18]. As the main bearers and executors of life activities, proteins are directly involved in the response of plants to Al stress. The genome of organisms is usually stable and remains unchanged during growth and development stages and during various physiological and pathological stages. In contrast, the proteome phenotype constantly changes, and different external environmental changes induce changes in the gene expression products, produce (differentially expressed proteins) DEPs, regulate the production and activity of enzymes, and cause changes in the secondary metabolites of organisms. Simultaneously, the transcription and translation of proteins directly determine the structure and function of plant cell membranes, cytoplasm, cytoskeleton, and other organelles composed of proteins. The differential expression of proteins as the key mediator roles of plants in response to environmental stresses can reflect the changes in plant physiological indicators when they are subjected to stresses, while protein synthesis and catabolism are also regulated by physiological changes in plants. In the field of bioinformatics, we used proteomics to study the response mechanism of *Camellia drupifera* to different concentrations of Al stress, and the results showed that *C. drupifera* mainly resisted Al toxicity through metabolic pathways such as energy and carbohydrate metabolism, amino acid synthesis and metabolism, alkaloid synthesis, protein processing, and porphyrin and chlorophyll metabolism under Al stress [19]. Then, by analyzing the function of DEPs to identify the key metabolites and verifying the function of their genes, it was confirmed that flavonoid compounds play a key role in the response of *C. drupifera* to Al stress [20].

*Camellia semiserrata*, an important woody edible oil tree species, is a tree or shrub-like plant in the genus *Camellia* of the family *Camelliaceae*. The tree is concentrated in the hilly areas of South China, located in the low hills along the Suijiang and Xijiang River Basins that border the two provinces of Guangdong and Guangxi. With its large bright red flowers, large fruits, and seed kernels with high oil contents, the plant is an important woody edible oil tree species in South China. The tea oil extracted from its seed kernels is rich in monounsaturated fatty acids, vitamins, and unique active substances (e.g., squalene, flavonoids, camellia glycosides, camellia saponins), which have certain market value and research prospects. In addition, the *C. semiserrata* tree is tall, with a beautiful tree shape, dark green glossy leaves, spring flowers, and autumn fruit of excellent garden species. Numerous studies have examined the inhibition of *C. semiserrata* growth and development due to Al toxicity; however, the mechanism underlying the proteomic response of *C. semiserrata* under Al stress remains unclear. For this reason, in this study, we used two-year-old *C. semiserrata* seedlings as the test material for a pot experiment and investigated their physiological response under different concentrations of Al stress. In addition, we conducted a comparative analysis of DEPs under aluminum stress using 4D label-free technology to summarize the response mechanism of *C. semiserrata* under aluminum stress at the physiological and proteomic levels. The technique of 4D proteomics is based on the three dimensions of 3D separation, i.e., retention time, mass-to-charge ratio (*m*/*z*), and ion intensity, and adds a fourth dimension, the separation of ion mobility, and dramatically improves the scanning speed and detection sensitivity, which brings about a comprehensive enhancement of the performance of proteomics in terms of identification depth, detection cycle, and quantitative accuracy.

## 2. Materials and Methods

### 2.1. Plant Materials

The oil tea variety used for the experiment was *C. semiserrata* from Xiaokeng Forest Farm (113°49′39.76″, 24°42′26.54″), Shaoguan City, Guangdong Province, China. In March 2022, healthy and identically growing 2-year-old live seedlings were transplanted into plastic pots (pot size 18 cm bottom diameter, 16 cm height, 23 cm top diameter) for cultivation. Subsequently, the seedlings were subjected to normal maintenance and management, and after 2 months of normal management, 15 healthy and basically uniform growth plants (divided into 5 groups, with 3 pots in each group) were selected and placed in a greenhouse at a temperature of 26 °C and a humidity of 60% to ensure the healthy growth of the seedlings of *C. semiserrata*.

### 2.2. Aluminum Stress Treatments

The experiment was set as control (clear water, CK) and aluminum treatment groups (1, 2, 3, and 4 mmol/L AlCl_3_-6H_2_O, analytically pure); three seedlings were set for each treatment. During the treatment period, *C. semiserrata* seedlings were transferred to an artificial climate chamber, in which the daytime temperature was set at 28 ± 2 °C, the night temperature was set at 25 ± 2 °C, the light intensity was adjusted to 600 µmol·m^−2^·s^−1^, the relative humidity was approximately 75%, and the solution was watered at intervals of 2 d with 200 mL each time. After 4 weeks of aluminum stress, the leaves were gently wiped with paper towels, photosynthesis was measured, and the leaf samples were collected three days after measuring photosynthesis; the samples were collected from 9:30 to 10:00, from the top to the bottom of the third to the fifth leaf. After collection, the leaves were individually wrapped in precooled 10 mL centrifuge tubes and immediately placed in liquid nitrogen to be transported to the laboratory and refrigerated at −80 °C. These samples were used for physiological experiments and protein analysis experiments of *C. semiserrata* leaves.

### 2.3. Physiological Measurements

Photosynthetic parameters, antioxidant enzyme activities, MDA, and osmoregulatory substance content were determined in each treatment group. The P*_n_*, T*_r_*, C*_i_*, and G*_s_* of plants were measured on a sunny day using a Li-6800 photosynthesis system (USA) with the light intensity set at 1200 μmol/(m^2^·s). SOD activity was determined via colorimetric colorimetry at 560 nm using nitroblue tetrazolium (NBT) photoreduction [21], POD activity was determined via colorimetric colorimetry at 470 nm using guaiacol as a substrate at 1 min intervals [22], and CAT activity was determined via colorimetric colorimetry at 405 nm using H_2_O_2_ as a substrate [23]. The MDA content was determined using the thiobarbituric acid (TBA) method with colorimetric measurements at 532 nm and 600 nm [24]. The absorbance values of soluble sugars were determined colorimetrically at 620 nm using the anthrone method [25], soluble proteins were determined colorimetrically at 595 nm using the Bradford method [26], and free proline was extracted with sulfosalicylic acid, followed by ninhydrin, and then determined via extraction with toluene and colorimetry according to the method of standard curve preparation [27]. All the physiological and biochemical indexes obtained were determined in three replicates.

### 2.4. Protein Extraction and Concentration Determination

After 1 g of leaf samples was weighed, the leaf samples were thoroughly ground into powder in liquid nitrogen. We added 1 mL of extraction solution and mixed well. An equal volume of phenol-Tris-HCl (7.8) saturated solution was added and mixed at 4 °C for 30 min, during which time it was shaken several times to mix, and then centrifuged at 4 °C, 7100 rpm, for 10 min to collect the phenol upper layer. We added 5 times the volume of pre-cooled 0.1 M ammonium acetate-methanol solution, precipitated the mixture overnight at −20 °C, centrifuged it at 4 °C, 12,000 rpm, for 10 min, and collected the precipitate. We added 5 times the volume of pre-cooled methanol to wash, mixed lightly, centrifuged the mixture at 4 °C, 12,000 rpm, for 10 min, collected the precipitate and repeated once. We repeated steps 7 and 8 twice with acetone instead of methanol to remove methanol sufficiently. We centrifuged the mixture at 4 °C, 12,000 rpm, for 10 min and collected the precipitate. It was then dried at room temperature and dissolved in sample lysate for 3–5 min at room temperature. The solution was centrifuged at 12,000 rpm for 10 min at room temperature, the supernatant was removed, and the solution was centrifuged again to remove the supernatant. The supernatant is the total protein solution of the sample. The protein concentration was determined using the Bradford method. The Bradford working reagent was composed of 0.01% (*w*/*v*) G250, 8.5% phosphoric acid, and 4.75% ethanol, which was preserved in a brown reagent bottle at room temperature for three months. Part of the protein solution was taken to be tested and diluted with ultrapure water (to prevent excessive concentration from exceeding the working range of the standard curve). BSA standard protein solutions with concentrations of 1.0, 0.8, 0.4, 0.2, and 0.1 mg/mL were prepared. Ten microlitres of BSA standard protein solution was added to each well of a 96-well plate, and 90 μL of water was added to bring the volume to 100 μL. Two microliters of protein solution was added to a 96-well plate with three wells for each sample, and the volume of the protein solution was added to 100 µL. Then, 100 µL of preconfigured working reagent was added to each well, and the mixture was maintained for 10 min. The absorbance value (wavelength 595 nm) was determined with an enzyme labeling instrument. Then, the standard curve was calculated according to the known concentration and absorbance value of the standard protein solution, and by substituting the absorbance value of the sample to be measured, the protein concentration value was calculated.

### 2.5. SDS-PAGE Electrophoresis

A total of 10 μg proteins of each sample was acquired and separated with 12% SDS-PAGE gel. Then, it was stained and washed for 15 min. Finally, the stained gel was scanned with an automatic digital gel image analysis system (Tanon 1600)

### 2.6. FASP Enzymatic Digestion, 4D-Label-Free Chromatographic Conditions and Mass Spectrometric Conditions

According to the results of protein quantification, one hundred micrograms of protein extract was subjected to 120 μL of reducing buffer (10 mM DTT, 8 M urea, 100 mM TEAB, pH 8.0) in a 10K ultrafiltration tube. The solution was incubated at 60 °C for 1 h, and IAA was added to the solution at a final concentration of 50 mM in the dark for 40 min at room temperature. Then, the solutions were centrifuged on the filters at 12,000 rpm for 20 min at 4 °C, and the flow-through solution was discarded from the collection tube. One hundred microliters of 300 mM TEAB was added to the solutions and centrifuged at 12,000 rpm for 20 min, and this step was repeated twice. After the washing step, the filter units were transferred into new collection tubes, and 100 μL 300 mM TEAB and 3 µL sequencing-grade trypsin (1 μg/μL) were added in each tube. Then, the solutions were incubated for digestion at 37 °C for 12 h. Finally, the collected digested peptides were centrifuged at 12,000 rpm for 20 min. Then, 50 μL 200 mM TEAB was added, and the samples were centrifuged again. The solutions were collected and lyophilized.

### 2.7. SOLA™SPE96 Desalting Plate

The solutions were added 1% to each sample to adjust the pH value to 7. The digested peptides were desalted with a SOLA™ SPE 96-plate Column. Firstly, the column was washed with 200 μL methanol 3 times, followed by 200 μL H_2_O 2–3 times. The samples were loaded on the column twice. Then, the column was washed with 5% methanol/H_2_O 3 times. Finally, the peptides were eluted with 150 μL 100% methanol 3 times and were lyophilized.

### 2.8. Liquid Chromatography

Nanoflow reversed-phase chromatography was performed on a nanoElute liquid chromatography system (Bruker Daltonics, Billerica, MA, USA). Peptides were separated in 90 min at a flow rate of 300 nL/min on a 25 cm × 75 μm column (1.6 μm C18, ionopticks). Mobile phases A and B were 0.1 vol% formic acid solution and Acetonitrile (ACN) with 0.1 vol% formic acid, respectively. The total run was 90 min (0~75 min, 2–22% B; 75~80 min, 22–37% B; 80~85 min, 37–80% B; 85~90 min, 80% B).

### 2.9. LC–MS/MS Analysis

Liquid chromatography was coupled online to a hybrid TIMS quadrupole TOF mass spectrometer (Bruker timsTOF Pro) via a CaptiveSpray nanoelectrospray ion source. The capillary voltage was 1.4 kV, the dry gas temperature was 180 °C, and the dry gas flow rate was 3.0. L/min. The dual TIMS analyzer was operated at a fixed duty cycle close to 100% using equal accumulation and ramp times of 100 ms. We performed DDA in PASEF mode with 10 PASEF scans per topN acquisition cycle. The full MS scan range was set from 100 to 1700 *m*/*z*. The ion mobility range was 0.6–1.6 vs./cm^2^, and the collision energy range was 20–59 eV.

### 2.10. Database Search

The LC–MS/MS raw data were imported into MaxQuant (Version 1.6.17.0) for labeling-free quantification analysis, and the search engine was Andromeda. The database used in this study is uniprot-*C. sinensis*-4442-2022.5.26.fasta (https://www.UniProt.org/, accessed on 26 May 2022). The main parameters were set as follows:
**Item****Value**FDR0.01Missed cleavage2Fixed modificationCarbamidomethyl(C)Variable modificationOxidation (M), Acetyl (Protein N-term)Decoy database patternReverseEnzymeTrypsinFirst search peptide tolerance20 ppmMain search peptide tolerance10 ppmDatabaseUniProt-*Camellia sinensis*-4442-2022.5.26.fasta

### 2.11. Statistical Analysis

Each experiment was repeated 3 times, and the data were recorded on Excel 2013 software. Mean, variance, standard deviation, and the analysis of significant differences between groups were performed using SPSS 21.0 software (SPSS Institute, Inc., Chicago, IL, USA), and on the basis of the obtained plausible proteins, 2 criteria were selected for the next screening of the differences between the proteins, the treatment group, and the control group. The screening conditions for DEPs were fold change ≥ 1.2 or fold change ≤ 1/1.2 and *p* value < 0.05. Compliance with this condition indicated that the differences between the screened DEPs were significant, and if the FC < 2, the expression of the proteins was considered undifferentiated. Proteins identified in different groups were used to generate a heatmap using TBtools v1.112 (https://github.com/CJ-Chen/TBtools/tags, accessed on 23 September 2023) based on their expression level differences. The Gene Ontology (GO) database (https://geneontology.org/, accessed on 23 September 2023) and Kyoto Encyclopedia of Genes and Genomes (KEGG) pathway database (http://www.genome.ad.jp/kegg/, accessed on 1 October 2023) were used for GO functional annotation of differential proteins and analysis of the metabolic pathways involved to obtain information on the biological functions, biological processes involved, and cellular localization of the differential proteins. Using the UniProt database (http://www.uniprot.org, accessed on 1 October 2023), the functions of all identified proteins were determined via GO and KEGG analyses, and the proteins were categorized according to their primary functions. Finally, the STRING-DB (http://string-db.org/, accessed on 7 October 2023) protein interaction database (selecting *C. sinensis*) was used to analyze the comparative and DEP interactions. Visualization of the data was performed using Python v3.12.0 (https://www.python.org/downloads/release/python-3120/, accessed on 7 October 2023).

## 3. Results

### 3.1. Plant Morphological Changes

**Figure 1:**
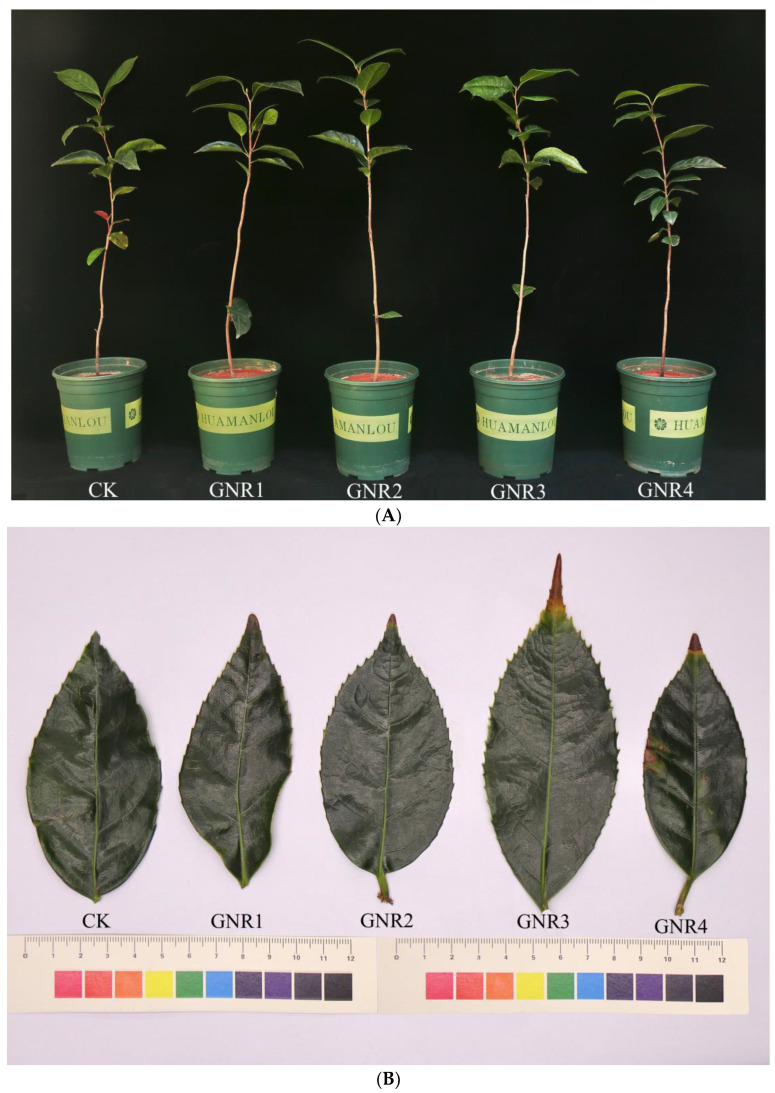
(**A**) Effects of aluminum stress on plant growth status. The five pots of seedlings from left to right indicate control plants, plants under 1 mmol/L aluminum stress, plants under 2 mmol/L aluminum stress, plants under 3 mmol/L aluminum stress, and plants under 4 mmol/L aluminum stress. (**B**) Effects of aluminum stress on plant leaf morphology. Corresponding to the aluminum treatment concentrations for the five seedlings in (**A**).

When *C. semiserrata* seedlings were subjected to aluminum stress, their growth was inhibited as the concentration of aluminum stress increased; in addition, the tips of the leaves turned yellow and the leaves curled or the whole leaf was removed. Our results showed that plants grew well under normal growth conditions (control group), whereas plants in treatment groups (3 mmol/L and 4 mmol/L) with high concentrations of Al stress showed more yellowing at the tips of apical leaves compared to the control group (Figure 1A,B). At 1 mmol/L and 2 mmol/L, the plants grew well, with clear apical buds visible to the naked eye and normal leaf morphologies; at 3 mmol/L and 4 mmol/L, the growth of the plants slowed, the growth and development of the apical buds were inhibited, and the edges of the leaf blades appeared to be curled and scorched (Figure 1A,B).

### 3.2. Photosynthetic Parameter Changes

**Figure 2:**
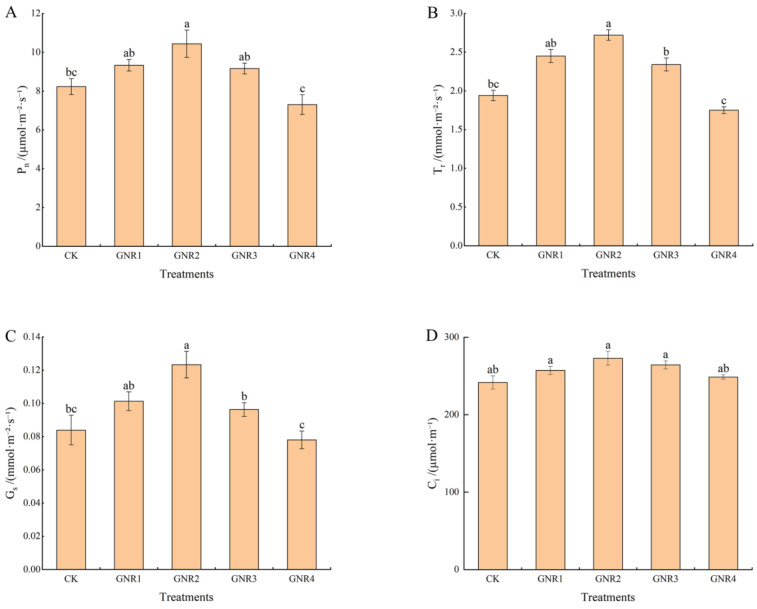
Effects of aluminum stress on plant photosynthesis. Different small letters indicate significant differences at *p* < 0.05. (**A**) net photosynthetic rate; (**B**) transpiration rate; (**C**) stomatal conductance; (**D**) intercellular CO_2_ concentration.

Under the effect of aluminum stress, the P*_n_*, T*_r_*, G*_s_*, and C*_i_* of *C. semiserrata* seedlings incrementally increased then decreased with increasing aluminum stress concentration. Compared with CK, P*_n_*, T*_r_*, and G*_s_* were higher at aluminum stress concentrations of 1 mmol/L, 2 mmol/L, and 3 mmol/L and reached the maximum value at 2 mmol/L; the differences between 1 mmol/L and 3 mmol/L were not significant (*p* < 0.05), while the differences were more significant (*p* < 0.05) at a concentration of 4 mmol/L. P*_n_*, T*_r_*, and G*_s_* were the lowest at this time, decreasing by 11.3%, 9.8%, and 7%, respectively, compared with that of CK. With the increase in aluminum stress, C*_i_* changed the least. Compared with CK, C*_i_* was higher at aluminum stress concentrations of 1 mmol/L, 2 mmol/L, and 3 mmol/L; there was no significant difference among the three, and C*_i_* declined when the concentration was elevated to 4 mmol/L; however, the value was slightly higher than that of CK (Figure 2). For detailed photosynthetic parameter data, please see Table S1.

### 3.3. Changes in Physiological Response

**Figure 3:**
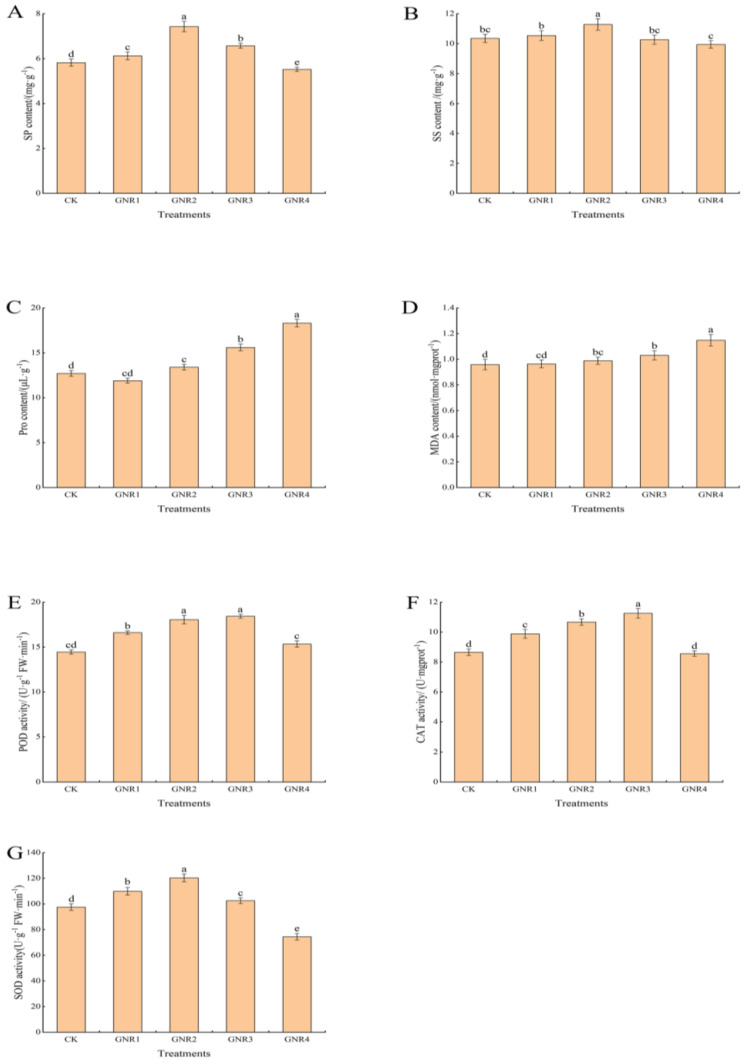
Effects of aluminum stress on plant physiological performance. Different small letters indicate significant differences at *p* < 0.05. (**A**) soluble protein (SP) contents; (**B**) soluble sugar (SS) contents; (**C**) proline (Pro) contents; (**D**) malondialdehyde (MDA) contents; (**E**) peroxidase (POD) activity; (**F**) catalase (CAT) activity; (**G**) superoxide dismutase (SOD) activity.

Under the influence of aluminum stress, different degrees of physiological and biochemical responses also occurred in the leaves of *C. semiserrata* seedlings. SP is an important indicator of enzyme system stability in plant cells, while SS is among the important osmoregulatory substances in the plant body. As shown in Figure 3A,B, the content of SP and SS exhibited the same trend in this experiment, i.e., decreasing with the increase in aluminum stress concentration. Compared to CK, their content was higher in the three treatment groups GNR1, GNR2, and GNR3; however, it was significantly reduced in the GNR4 treatment group; the contents of both were highest at the concentration of 2 mmol/L, 1.3 times and 1.1 times that of the control treatment, respectively. When the stress concentration rose to 4 mmol/L, the SP content decreased sharply, indicating that the synthesis of leaf protein was significantly inhibited. The SS content also decreased at this concentration, but there was no significant difference compared with the control. Pro and MDA play important roles in plant responses to various adverse conditions. As shown in Figure 3C,D, the calibration trends of Pro and MDA contents in the leaves of *C. semiserrata* were similar, with a general upward trend, and reached the maximum value at 4 mmol/L. Compared with CK, the contents of Pro and MDA were increased by 19.8% and 39.8%, respectively. POD, CAT, and SOD belong to the enzyme system of the plant antioxidant system, which can play important roles in plants under oxidative stress. The three antioxidant enzyme activities showed similar trends in this experiment, increasing and then decreasing with increasing stress concentration, but the nodes at which the decreases occurred were different (Figure 3E–G). POD and CAT activities gradually increased at stress concentrations of 0–3 mol/L and were most active at 3 mmol/L, with 27.6% and 30.0% enhancement, respectively, compared with CK. Then, the activities of the two enzymes significantly decreased when the concentrations were increased to 4 mmol/L, and the values were not significantly different from those of CK. The activity of SOD, on the other hand, increased at 0–2 mol/L, with a maximum value of 23.3% enhancement compared with that in the CK group. SOD activity decreased rapidly at subsequent treatment concentrations and was lowest at 4 mol/L, differing significantly from the control with a 23.7% reduction in activity.

### 3.4. Principal Component Analysis (PCA)

**Figure 4:**
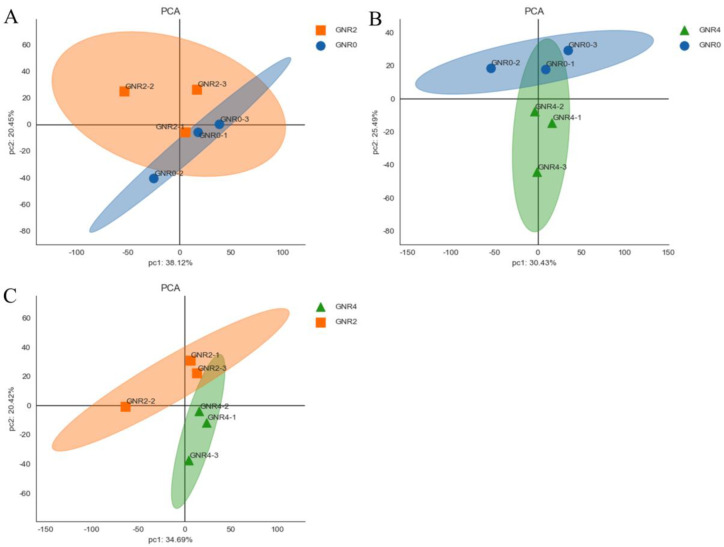
PCA score of *C. semiserrata* leaves. (**A**) GNR2/GNR0; (**B**) GNR4/GNR0; (**C**) GNR4/GNR2.

PCA of the leaves of *C. semiserrata* can initially reveal the overall metabolic differences between samples among the groups and more intuitively illustrate the size of the variability between samples within the groups. As shown in Figure 4, the raw data obtained from the principal component analysis using trusted protein expression were well separated and presented in the two principal components of PC1 and PC2.

### 3.5. Protein Identification Results

CK (GNR0), GNR2, and GNR4 *C. semiserrata* leaves were used for protein identification. After each sample was detected with LC–MS/MS and searched in libraries, we identified 26,028 peptides, 14,768 unique peptides, and a total of 3960 proteins (Table 1). The qualitative and quantitative results of the proteins are shown in Tables S2 and S3.

#### Screening of DEPs

**Figure 5:**
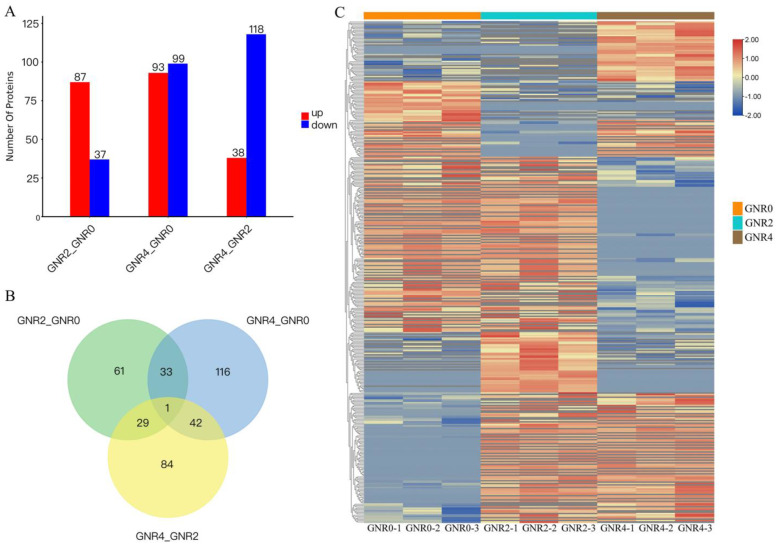
DEP analysis in comparison groups of GNR2/GNR0 and GNR4/GNR0. (1) (**A**) Statistical chart of DEPs in leaves of *C. semiserrata*; (**B**) shows the DEP Venn diagram; (**C**) shows the heatmap of clustering analysis of differential comparison groups. (2) (**C**) clustering heatmap, red indicates high expression protein, blue indicates low expression protein, and each row indicates the expression of each protein in different groups.

The GNR0 group, GNR2, and GNR4 were compared in a two-by-two comparison (i.e., GNR2/GNR0, GNR4/GNR0, GNR4/GNR2); Foldchange ≥ 1.2 or Foldchange ≤ 1/1.2 and *p* value < 0.05 were set for screening of DEPs. As shown in Figure 5A, and compared with the GNR0 group, 124 DEPs were present in *C. semiserrata* leaves of GNR2, of which 87 DEPs were upregulated and 37 DEPs were downregulated. There were 192 DEPs in the leaves of GNR4, of which 93 DEPs were upregulated and 99 DEPs were downregulated. A total of 156 DEPs were identified in GNR4 leaves compared to GNR2 leaves, of which 38 DEPs were upregulated and 118 were downregulated. In the comparison groups of GNR2/GNR0 and GNR4/GNR0, a total of 34 coacting DEPs were found, and these proteins may occupy a leading position in the resistance of *C. semiserrata* seedlings to aluminum stress (Figure 5B). The expression of each protein can be clearly seen in Figure 5C and Table S3.

### 3.6. GO Enrichment Analysis of DEPs under Aluminum Stress

**Figure 6:**
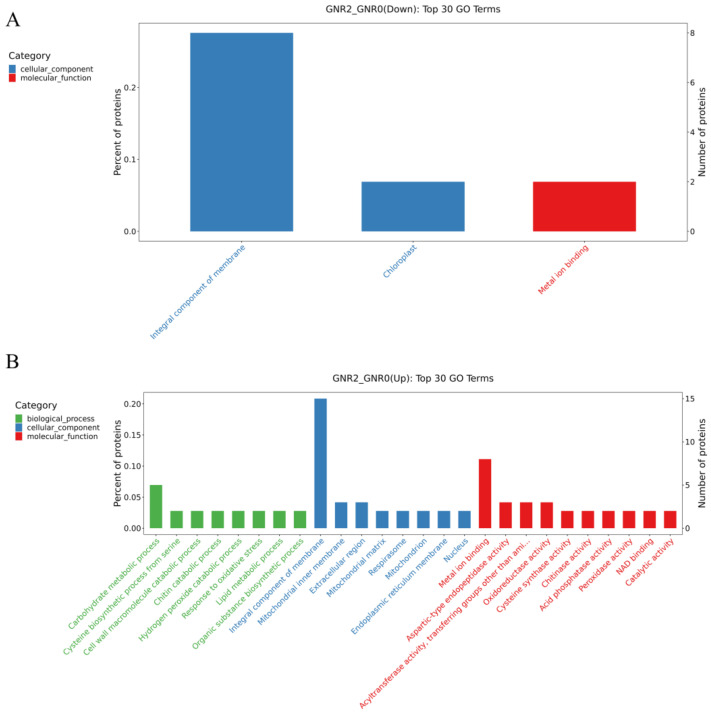

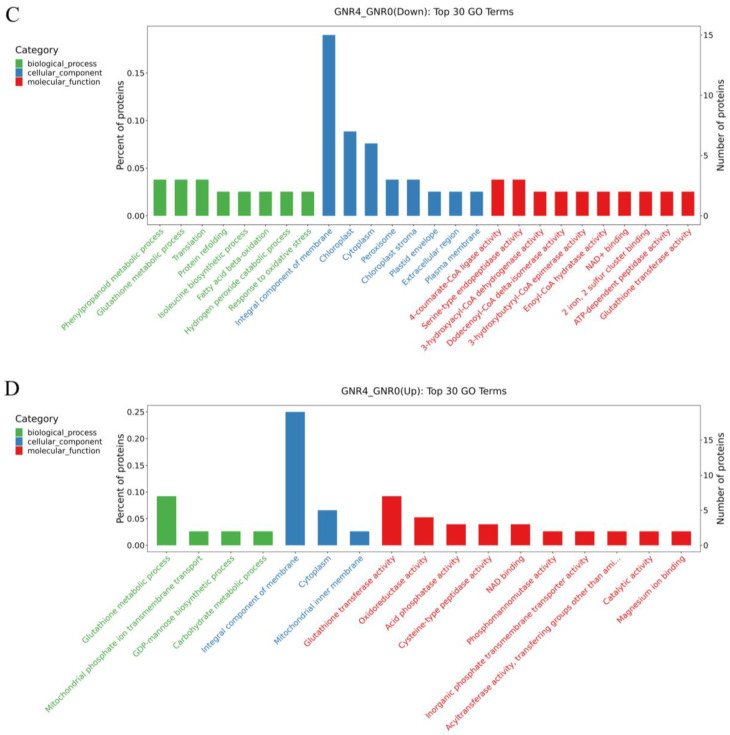
GO enrichment analysis of DEPs in GNR2/GNR0 and DR4w/CK. (1) (**A**) indicates the DEPs downregulated by GNR2/GNR0; (**B**) indicates the DEPs upregulated by GNR2/GNR0; (**C**) indicates the DEPs downregulated by GNR4/GNR0; (**D**) indicates the DEPs upregulated by GNR4/GNR0. (2) The x-coordinate in the graph is the name of the GO entry, and the y-coordinate is the number of proteins in the corresponding entry and their percentage.

To clarify the functional annotation and classification of DEPs, Blast 2 GO v6.0 (https://www.blast2go.com/, accessed on 1 October 2023) was used to perform GO functional annotation of the screened DEPs, which contained biological process (BP), cellular component (CC), and molecular function (MF). In this study, we viewed all the identified proteins as a screening library and screened the GO entries with ListHits greater than 1 in the three ontologies under different Al stress treatments, with 10 entries each sorted in ascending order according to the −log10 *p* value corresponding to each entry. A bar chart for the top 30 GO function enrichment analyses using data visualization was formed with the results obtained (Figure 6).

Figure 6A,B shows the comparison of GNR2 with GNR0, in which downregulated DEPs were enriched in the following GO terms: cellular components and molecular functions, in which ‘integral component of membrane’ (GO:0016021) and ‘chloroplast’ (GO:0009507) belong to the cellular component GO term, and ‘metal ion binding’ (GO:0046872) belongs to the molecular function GO term. For the upregulated DEPs, the most enriched term in the cellular component category was ‘integral component of membrane’ (GO:0016021), followed by the molecular function GO term ‘metal ion binding’ (GO:0046872). Therefore, these proteins might play a significant role in alleviating aluminum stress in safflower *C. semiserrata* leaves. Figure 6C,D illustrates the GO enrichment analysis of DEPs in the GNR4/GNR0 group. The DEPs that were significantly enriched in GO terms among the downregulated DEPs included ‘integral component of membrane’ (GO:0005975), which belonged to the cellular component, and there were 15 proteins involved in these GO terms. Furthermore, high enrichment results were also obtained for ‘chloroplast’ (GO:0009507) and ‘cytoplasm’ (GO:0005737) in the cellular component. In the biological process GO term, high GO enrichment results were obtained for ‘phenylpropanoid metabolic process’ (GO:0009698), ‘glutathione metabolic process’ (GO:0006749), and ‘translation’ (GO:0006412), and the significantly enriched molecular function GO terms included ‘4-coumarate-CoA ligase activity’ (GO:0016207) and ‘serine-type endopeptidase activity’ (GO:0004252). For the upregulated DEPs, the significantly enriched cellular component GO term was ‘integral component of membrane’ (GO:0016021). In addition, ‘glutathione metabolic process’ (GO:0006749) was enriched in biological process terms. Notably, ‘oxidoreductase activity’ (GO:0016491) in molecular function was upregulated in the GNR4/GNR0 group.

### 3.7. KEGG Enrichment Analysis of DEPs under Aluminum Stress

To further clarify the metabolic processes involved in differential proteins, pathway analysis of differential proteins was carried out using the KEGG database to analyze the enrichment in biological pathways and functions concentrated in these DEPs. In this study, KEGG enrichment analysis can more easily show the differential metabolism under different concentrations of Al stress in *C. semiserrata*.

**Figure 7:**
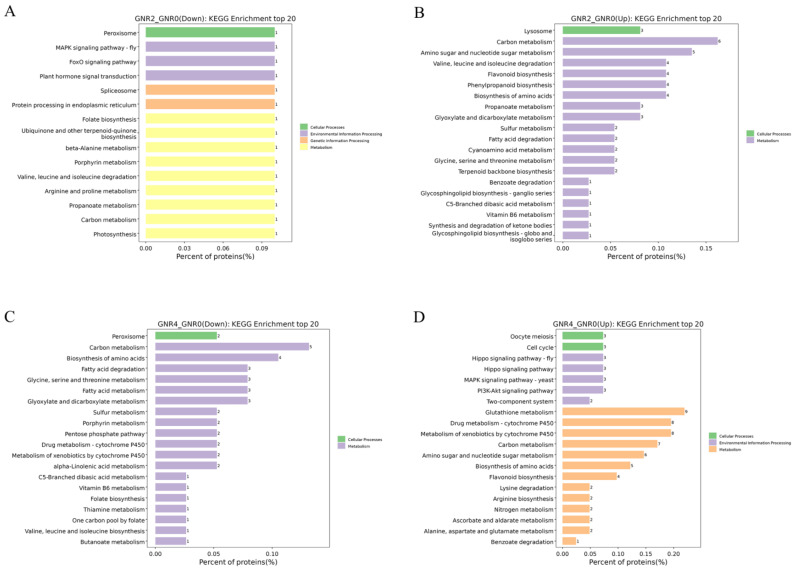
KEGG enrichment analysis of DEPs in GNR2/GNR0 and GNR4/GNR0. (1) (**A**) indicates the DEPs downregulated by GNR2/GNR0; (**B**) indicates the DEPs upregulated by DR2w/CK; (**C**) indicates the DEPs downregulated by GNR4/GNR0; (**D**) indicates the DEPs upregulated by GNR4/GNR0. (2) The *x*-axis percentage of proteins is the percentage of protein expression, and the *y*-axis is the pathway information of the top 20 DEPs.

Figure 7A,B shows the KEGG metabolic pathways for the DEPs in “GNTR2/GNR0”. Among the downregulated metabolic pathways, only fifteen differential metabolic pathways were enriched, of which nine belonged to metabolism, three belonged to environmental information processing, two belonged to genetic information processing, and one belonged to cellular processes. Each KEGG term contained only one DEP. Among the upregulated metabolic pathways, those belonging to Metabolism were significantly increased, and more DEPs were mainly enriched in ‘Carbon metabolism’, ‘Amino sugar and nucleotide sugar metabolism’, ‘Flavonoid biosynthesis’, ‘Phenylpropanoid biosynthesis’, ‘Phenylpropanoid biosynthesis’, and other metabolic pathways. These metabolic pathways are mostly related to energy synthesis and defense mechanisms against environmental stresses, suggesting that plants under Al stress begin to perform a series of complex biochemical reactions to resist this stress.

With a further increase in Al stress, KEGG pathways in the “GNTR4/GNR0” group showed interesting changes compared to those in the “GNTR2/GNR0” group. The KEGG pathways in the “GNTR4/GNR0” group showed interesting changes compared with the “GNTR2/GNR0” group. As shown in Figure 7C,D, part of ‘Carbon metabolism’ became a downregulated metabolic pathway, along with ‘Biosynthesis of amino acids’, ‘Fatty acid metabolism and degradation’, ‘Glyoxylate and dicarboxylate metabolism’, etc. More DEPs in the metabolic pathways upregulated by the GNTR4/GNR0 group were involved in the processes of metabolism, such as ‘glutathione metabolism’, ‘drug metabolism—cytochrome P450’, ‘metabolism of xenobiotics by cytochrome P450’, and ‘carbon metabolism’, and other KEGG pathways were significantly enriched. In addition, upregulated metabolic pathways were enriched in ‘Hippo signaling pathway’, ‘MAPK signaling pathway—yeast’, ‘PI3K-Akt signaling pathway’, and other pathways in the Environmental Information Processing PI3K-Akt signaling pathway’, and ‘Cell cycle’ and ‘Oocyte meiosis’ pathways in Cellular Processes were also upregulated.

### 3.8. Identification of Protein–Protein Interaction (PPI) Networks among DEPs

**Figure 8:**
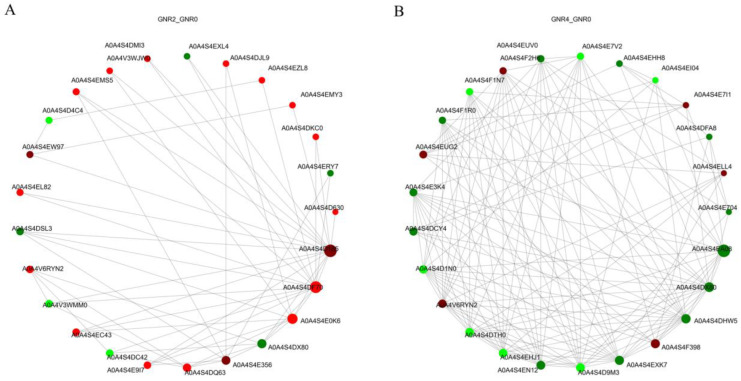
PPI analysis of DEPs in GNR2/GNR0 (**A**) and GNR4/GNR0 (**B**). The circles in the figure represent DEPs, red represents upregulated DEPs, and green represents downregulated DEPs; the size of the circles represents the level of connectivity, with larger circles indicating higher connectivity.

Protein interactions in organisms are an extremely complex process, including synergistic and antagonistic effects among proteins. To analyze the interactions among DEPs in the leaves of *C. semiserrata*, DEPs under different concentrations of aluminum stress treatments were subjected to differentially expressed protein interaction network analysis based on the STRING database (https://string-db.org/, accessed on 7 October 2023). The top 25 nodes ranked by node connectivity were visualized using Python v3.12.0, protein IDs were used for display to draw PPI interaction network graphs, and the results are shown in Figure 8A,B. Comparison of GNTR2/GNR0 indicated that 35 DEPs were involved in the protein interaction network under aluminum stress, among which Aconitase_C domain-containing protein (A0A4S4D185) had 19 reciprocal nodes, ECH_2 domain-containing protein (A0A4S4DF70) had 16 nodes, followed by NmrA domain-containing protein (A0A4S4E0K6) with 11 nodes and cysteine synthase (A0A4S4E356) with 7 nodes. (A0A4S4DX80 with eight nodes was an Uncharacterized protein). For the comparison of GNTR4/GNR0, a total of 64 DEPs were involved in the protein interaction network under aluminum stress. The protein with the most nodes was 4-coumarate-CoA ligase (A0A4S4EA08), which had 37 nodes, followed by peptide deformylase (A0A4S4DHW5) with 20 nodes and hydroxymethylbilane synthase (A0A4S4D9M3) and chromodomain-containing protein (A0A4S4EN12), which each had 18 nodes, and the top 25 DEPs all had more than 8 node connections.

### 3.9. Screening of Key DEPs in Leaves of C. semiserrata Seedlings under Aluminum Stress

Based on the results of GO and KEGG enrichment analysis, the significantly different entries or pathways in the GNR2/CK and GNR4/CK comparison groups were analyzed. The DEPs with more participating entries or pathways in the comparison groups were selected, focusing on the DEPs that continued to play a role in both comparison groups. Then, these DEPs were categorized according to function under KEGG, and 73 DEPs (including 42 upregulated and 31 downregulated DEPs) were further screened. Under aluminum stress treatment, DEPs in the leaves of *C. semiserrata* were mainly enriched in glutathione metabolism (W6ACL7, A0A7J7HB73, A0A7J7HD86, etc.), carbon metabolism (A0A7J7H0N1, T1WY53, D3YN47, etc.), photosynthesis (A0A067YPP4, A0A7J7GLK3, A0A7J7I432, etc.), and flavonoid biosynthesis (H9AZQ2, Q6DV45, A0A6N0C6H2, etc.). Some key proteins are shown in Table 2. Based on the metabolic pathways of KEGG, the known proteins can be roughly classified into the following four categories:

## 4. Discussion

### 4.1. Physiological Response of C. semiserrata Leaves under Aluminum Stress

Photosynthesis provides most energy needed for plant growth and development, supports the synthesis of organic matter, and supports the release of oxygen. These processes support nearly all of the plant’s life activities. Studies have shown that the amount of photosynthesis products directly affects plant biomass [28]. In addition, the strength of photosynthesis has an important impact on plant resistance, so photosynthesis is often used as an important indicator to determine the growth status and resistance strength of plants [29]. In this study, we found that P*_n_*, T*_r_*, G*_s_*, and C*_i_* tended to increase and then decrease with increasing concentration of aluminum stress (0–4 mmol/L), possibly indicating that under certain conditions, low concentration of aluminum stress can promote photosynthesis in oil tea and that *C. semiserrata* exhibits a certain degree of resistance to aluminum stress. This result is consistent with the conclusion of Huang [13]. As the concentration continued to increase, P*_n_*, T*_r_*, and G*_s_* decreased significantly and C*_i_* decreased, but there was no significant difference between the groups; therefore, C*_i_* does not directly weaken photosynthesis under high concentrations of aluminum stress.

When plants are subjected to abiotic stresses, plant cells exhibit a range of physiological changes in response to environmental stresses. Osmoregulation is among the important mechanisms of plants in response to abiotic stresses, and this regulation helps maintain the osmotic stability of plant cells and mitigate the negative effects caused by environmental stresses [30,31]. In this study, SP and SS contents were higher in the GNR2 treatment group, indicating that the anabolic metabolism of SP and SS in the cells was enhanced under the 2 mmol/L aluminum stress concentration. In addition, the contents of SP and SS were elevated, which reduced the osmotic potential of the cells and maintained the stability of the cellular structure and function. The SP and SS contents decreased to different degrees after the concentration further increased, indicating that SP and SS synthesis was inhibited, and the ability of the cells to maintain osmotic pressure stability decreased. Under excess aluminum, the intracellular balance of free radical metabolism is broken, causing the accumulation of excess superoxide anion and hydrogen peroxide, leading to the destruction of the cell membrane structure and causing oxidative damage to plant cells [32,33]. Pro is well known as an osmoregulatory substance and is an important antioxidant that effectively scavenges ROS and maintains plasma membrane stability [34]; the accumulation of intracellular ROS can lead to the peroxidation of membrane lipids, which produces MDA and other products of peroxidation of cell membrane lipids. Therefore, the contents of Pro and MDA can represent the stress avoidance indicators of plants. In this study, the Pro and MDA contents generally showed an increasing trend with increasing concentration, especially at 2 mmol/L–4 mmol/L concentrations. This result indicates that *C. semiserrata* suffered severe membrane damage in response to aluminum stress, and the physiological functions of the leaves were affected. To resist the effects of this stress, plants often scavenge excess ROS by regulating the antioxidant enzyme system, which mainly consists of POD, CAT, and SOD; these enzymes act synergistically against oxidative stress and work together to scavenge intracellularly enriched hydrogen peroxide and superoxide anions, thus reducing or preventing oxidative damage to the plant [19,35]. In this experiment, the activities of POD, CAT, and SOD in *C. semiserrata* leaves showed an increasing and then decreasing trend with increasing stress concentration. Therefore, all three enzymes played a role in scavenging reactive oxygen species in the experiment and *C. semiserrata* exhibited a certain degree of aluminum tolerance. SOD reached its peak value at a concentration of 2 mmol/L, while POD and CAT peaked at 3 mmol/L, indicating that SOD exhibits a higher sensitivity to the aluminum stress environment in *C. semiserrata*. In addition, the activities of the three antioxidant enzymes decreased significantly at 4 mmol/L, indicating that the ROS accumulated in the cells of *C. semiserrata* leaves had exceeded the scavenging ability of the three enzyme-protecting enzymes, resulting in the inhibition of antioxidant enzyme activities.

### 4.2. Functional Analysis of DEPs in C. semiserrata Leaves under Aluminum Stress

#### 4.2.1. Photosynthesis-Related Proteins

Photosynthesis occurs in the green tissues of all plants and usually consists of two phases, light and dark reactions; these phases involve several reaction steps such as light absorption, electron transfer, photosynthetic phosphorylation, and carbon assimilation [36]. In this study, 22 DEPs were found to be involved in the regulation of photosynthesis. Five of the DEPs changed significantly in GNR2, sixteen DEPs changed in GNR4, and one additional DEP changed significantly in both GNR2 and GNR4. In GNR2, photosystem II D2 protein (A0A067YPP4), divinyl chlorophyllide α 8-vinyl-reductase (A0A7J7GLK3, DVR), thioredoxin domain-containing protein (A0A7J7I432), and CP12 domain-containing protein (A0A4V3WJ29) were downregulated, and the expression of glyceraldehyde-3-phosphate dehydrogenase (A0A7J7H8S9, GAPDH) and aspartate aminotransferase (A0A4S4EUG2, AspAT) was upregulated.

The photosystem II D2 protein is involved in the photosystem II process of light reactions and plays a role in key photosynthetic steps, such as the capture of light energy, electron transfer, and water catabolism [37]. DVR is an important reductase in the synthesis of chlorophyll a, as it reduces the two 8-vinyl groups in the precursor molecule of chlorophyll a to the 8-ethyl group to form chloroplast pigment a. Chlorophyll a also plays a role in photosynthesis by trapping light energy and contributing to electron transfer and ATP synthesis in photosynthesis [38]. Thioredoxin domain-containing proteins perform a variety of functions, which can not only regulate the activity of photosynthesis-related enzymes but also maintain the redox balance in chloroplasts through a series of redox reactions. CP12 domain-containing protein is involved in regulating the thioredoxin-mediated Calvin cycle, a dark reaction in photosynthesis, in which plants convert CO_2_ into organic compounds, such as glucose. CP12 domain-containing proteins can help maintain efficient photosynthesis by interacting with other enzymes and proteins and regulating the activity of enzymes in the Calvin cycle [39]. The expression of these proteins was downregulated, indicating that 2 mmol/L aluminum stress affects the light reaction, dark reaction, and chlorophyll synthesis processes in the leaves of *C. semiserrata*. These processes lead to a decreasing trend of chlorophyll content, affecting the photosynthetic rate. A further increase in the concentration of the stress may lead to a decrease in the rate of photosynthesis. In conjunction with the indicators of photosynthetic rate in the physiological experiments, photosynthesis also reaches the maximum value at GNR2. Among the upregulated DEPs, GAPDH exhibits complex physiological properties, as it catalyzes the production of glyceraldehyde-3-phosphate in the dark reaction of photosynthesis, which is a precursor for the synthesis of sugars, as well as other organic compounds; GAPDH also participates in the glycolytic pathway during cellular respiration, oxidizing glycerol 3-phosphate to glycerol 3-phosphate to produce ATP and release energy [40]. Furthermore, GAPDH plays an important role in plant stress tolerance. Zhao et al. showed that overexpression of GAPDH is beneficial to soybean tolerance to salt stress [41]. AspAT is involved in several biochemical reaction processes, including amino acid metabolism, nitrogen metabolism, and the tricarboxylic acid cycle process; AspAT mainly contributes to the synthesis of aspartic acid in photosynthesis, which plays a key role in carbon fixation and organic matter synthesis in the dark reaction process [42]. The expression of these two proteins was upregulated, indicating that the leaves of *C. semiserrata* actively respond to aluminum stress through a series of positive photosynthesis events.

Among the 17 DEPs that were significantly changed in GNR4, the expression of only 3 DEPs was upregulated, and the expression of the remaining 14 DEPs was downregulated. For these DEPs, the expression of DVR, which was downregulated in GNR2, was still downregulated in this process. Similarly, the expression of prolycopene isomerase (A0A4S4EUN1), which regulates carotenoid synthesis and metabolism, and hydroxymethylbilane synthase (A0A4S4D9M3), which regulates the biosynthesis pathway of heme and chlorophyll, was downregulated during this process. Therefore, the synthesis of chlorophyll and carotenoids was significantly inhibited during aluminum stress, which may be a main cause for the yellowing of *C. semiserrata* leaves after stress. Moreover, several proteins involved in NADPH production and carbon fixation in photosynthesis, such as ferredoxin (A0A7J7GPC2), Rieske domain-containing protein (A0A4S4E3K4), and D-3-phosphoglycerate dehydrogenase (HYC85_024613), were downregulated. Therefore, the light response and carbon sequestration capacity were severely affected, leading to a decrease in photosynthetic capacity. This trend is also consistent with the results of previous physiological experiments on photosynthesis.

#### 4.2.2. Flavonoid Biosynthesis-Related Proteins

Flavonoids are a class of secondary metabolites that regulate plant growth and development and play an important role in the resistance of plants to stress and adversity [43]. Our preliminary experiments demonstrated that flavonoid compounds play an important role in the resistance of *C. drupifera* to aluminum stress [20]. In this study, four DEPs were identified in leaves under 2 mmol/L aluminum stress. The expression of all DEPs was upregulated during stress, and all DEPs were involved in the anthocyanin biosynthesis pathway, are shown in Figure 8. Chalcone synthase 3 (H9AZQ2, CHS3), a key enzyme involved in flavonoid biosynthesis, catalyzes the conversion of 4-coumaroyl-Coa to chalcone, which is the precursor of various flavonoid compounds [44]. The upregulation of flavanone 3-hydroxylase (Q6DV45, F3H) expression further enhances the ability of chalcone to catalyze to dihydrokaempferol, which is an important intermediate in the synthesis of various flavonoids, including anthocyanins and proanthocyanidins [45]. Immediately thereafter, the expression of dihydroflavonol 4-reductase (A0A6N0C6H2, DFR) is also upregulated, resulting in the accelerated conversion of dihydrokaempferol to leucoanthocyanidin, which is also an important intermediate in the synthesis of anthocyanins and proanthocyanidins [45]. Amino acid transporter transmembrane domain-containing protein (A0A4S4DFF3) is mainly synthesized in plants as a UDP-glucosyl transferase (UGT). UGT is among the key enzymes involved in the regulation and control of anthocyanin metabolism, and flavonoid 3-O-glucosy transferase ultimately synthesizes anthocyanins by transferring and linking glucose from the UDP-glucose substrate to the C3 hydroxyl group of anthocyanins [46], which exhibit a strong antioxidant capacity in order to neutralize free radicals and reduce damage to plant cells from environmental-stress-induced oxidative stress [47].

**Figure 9:**
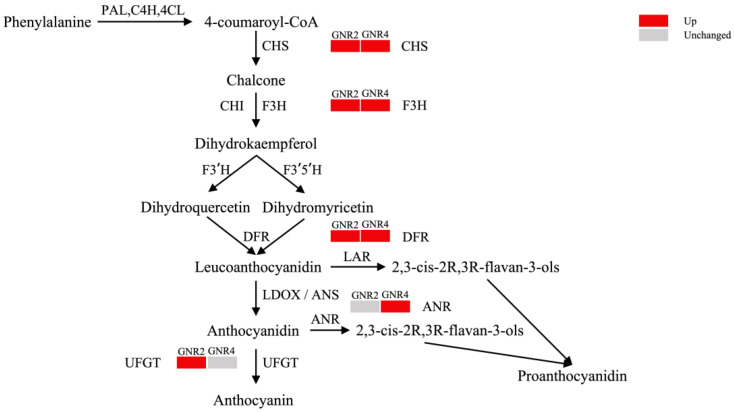
Mechanism of anthocyanin synthesis pathway response in *C. semiserrata* under Al stress.

Under aluminum stress at 4 mmol/L, four DEPs associated with flavonoid production were also detected, and their expression was upregulated compared with the control. Three of the DEPs, CHS3, F3H, and DFR, which were detected in GNR2, were detected at this time, and another GNR4-specific protein was anthocyanidin reductase (A0A481Y6T5, ANR2), which converts anthocyanidin to proanthocyanidin after a series of reactions. Proanthocyanidins also exhibit powerful antioxidant properties and can neutralize harmful free radicals, reduce oxidative damage, protect cellular structure and function, and help plants combat adversity-induced oxidative stress [48]. In our study, the expression of the three DEPs was consistently upregulated under different concentrations of stress, suggesting that *C. semiserrata* improves aluminum stress tolerance by increasing flavonoid biosynthesis under different aluminum stresses; these results correspond with findings from our study on flavonoid metabolism under aluminum stress in *C. drupifera* [20]. As the contents of proanthocyanidin and anthocyanins were positively correlated with the expression levels of ANR and UGT [49], we hypothesized that *C. semiserrata* resisted aluminum toxicity mainly by synthesizing anthocyanidins under 2 mmol/L stress; in contrast, proanthocyanidins were alleviated by synthesizing proanthocyanidins under 4 mmol/L stress, which may be a special mechanism through which *C. semiserrata* responds to different concentrations of aluminum stress (Figure 9).

#### 4.2.3. Antioxidant-Related Proteins

When plants are subjected to stress, the cells produce a large amount of ROS, and the serious accumulation of ROS causes irreversible damage to the structure and function of the cells. At this time, the cells establish certain defense mechanisms to resist the continuous production of ROS, and the antioxidant system is among the important defense mechanisms; then, the plants can regulate the antioxidant system to improve their tolerance to environmental stress [50]. The antioxidant system can be categorized into enzymatic and nonenzymatic systems [51], and the enzymatic system mainly consists of SOD, CAT, POD, APX, and GPX, while the nonenzymatic systems mainly consist of VC, GSH, polyphenols, flavonoids, etc. [52].

Through the proteomics data analysis in this study, three antioxidant-related DEPs were identified due to ROS changes in the leaves of aluminum-stressed plants at 2 mmol/L. The expression of peroxidase (A0A4S4EEB5, A0A4S4D1Y7) was upregulated, and the expression of superoxide dismutase (A0A4V3WMM0) was downregulated. ROS contain oxygen-containing compounds that are more reactive than oxygen molecules (O_2_), including superoxide radicals (O_2_^−^), hydrogen peroxide (H_2_O_2_), and hydroxyl radicals (OH^−^) [53]. O_2_^−^ is among the starting points of oxygen radical production, which is naturally produced in cells, while SOD directly converts O_2_^-^ into more stable H_2_O_2_ and O_2_ and prevents O_2_^−^ from triggering a chain reaction of oxidative reactions; thus, SOD is often considered the first line of defense against ROS, while CAT and POD are the second line of defense in eliminating ROS because they usually target and scavenge H_2_O_2_, which can further convert H_2_O_2_ to H_2_O and O_2_ [34,54]. In addition to its ability to scavenge ROS, POD participates in phenylpropanoid biosynthesis, phenylpropane is the precursor of lignin synthesis, and lignin is among the main components of the plant cell wall. Lignin helps maintain the structure and hardness of the cell wall to ensure normal intracellular physiological and biochemical reactions; in addition, lignin exhibits an antioxidant effect, helping to protect the leaves from the damage caused by oxidative stress [55]. Wang et al. showed that lignin plays a crucial role in aluminum tolerance in *Pinus massoniana* roots [56]. Under 2 mmol/L aluminum stress, the expression of POD increased while that of SOD decreased, indicating that POD plays an important role in scavenging ROS. In addition, the O_2_^−^ produced by oxidative stress in *C. semiserrata* leaves gradually exceeded the O_2_^−^ scavenging capacity of SOD, and further increasing the stress concentration may lead to a decrease in SOD activity, which is consistent with the physiological result that SOD activity peaked at 2 mmol/L.

In the leaves studied at 4 mmol/L in the experimental treatment, a total of ten DEPs were identified, including two peroxidases (POD1, POD5) and eight GST-family proteins, eight GST-family proteins with four glutathione transferases (HYC85_011867, HYC85_011872, TEA_030065, TEA_030058), three GST N-terminal domain-containing proteins (HYC85_011868, HYC85_006703, HYC85_013957), and one GST C-terminal domain-containing protein (HYC85_011877). In this study, the expression of POD1, POD5, and HYC85_011867 was downregulated, while the expression of other proteins was upregulated. Glutathione (GSH) is an important antioxidant nonenzymatic substance involved in plant resistance to oxidative stress [57]. GST family proteins are responsible for transporting GSH in and out of the cell and thus can effectively regulate the intracellular GSH concentration. The functions of GST family proteins are very broad, and the eight GST proteins identified play roles in KEGG pathways, such as drug metabolism—cytochrome P450, glutathione metabolism, metabolism of xenobiotics by cytochrome P450, and other KEGG pathways. Cytochrome P450 enzymes (CYP450) are the largest enzyme family in plant metabolism and exhibit a wide range of catalytic activities. The enzymes are mainly involved in various oxidative reactions in organisms [58]. Under GNR4 conditions, the downregulation of POD expression led to the weakening of phenylpropane biosynthesis and a reduction in lignin content. As *C. semiserrata* leaves might be more susceptible to oxidative stress and pathogens, it was hypothesized that the curling and yellowing of *C. semiserrata* leaves had a certain correlation with the reduction in lignin content. In addition, the expression of many GST family proteins was upregulated at this time, indicating that the accumulation of ROS had exceeded the scavenging capacity of antioxidant enzymes. Furthermore, *C. semiserrata* leaves were under severe stress and attempted to eliminate reactive oxygen species by synthesizing GSH, which might be a response mechanism for *C. semiserrata* to cope with the high concentration of aluminum stress; these findings correspond with our preliminary study on the proteomics results of *C. drupifera* under 4 mmol/L aluminum stress [19].

#### 4.2.4. Carbohydrate Metabolism-Related Proteins

Common carbohydrates include monosaccharides (glucose and fructose), disaccharides (sucrose), oligosaccharides, and polysaccharides and are the main source of energy for plants produced by photosynthesis [59]. The biochemical breakdown of these substances provides a large source of energy for plant growth and development. Carbohydrate metabolism is involved in all aspects of plant growth and development, and its metabolism also plays an important role in plant resistance to adverse stress [60].

In this study, 29 DEPs related to carbohydrate metabolism were identified in GNR2 vs. CK and GNR4 vs. CK. Compared with the control group, 14 carbohydrate metabolism-related DEPs were identified in the GNR2-treated group, among which 13 DEPs showed increased expression and 1 DEP showed decreased expression. In GNR4, 18 characterized DEPs were identified, of which 12 DEPs showed increased expression and 6 DEPs showed decreased expression. In addition, GDP-mannose-3’,5’-epimerase (M9RZ20), acetyl-CoA C-acetyltransferase protein (D3YN47) and glycine cleavage system H protein (A0A4S4EMY3) were upregulated in GNR2 and GNR4. Based on the functions of these proteins, nearly all the proteins are involved in multiple pathways of carbohydrate metabolism, among which acetyl-CoA C-acetyltransferase protein (D3YN47) can be involved in pyruvate metabolism, terpenoid backbone biosynthesis, synthesis and degradation of ketone bodies, etc. Acetyl-CoA C-acetyltransferase protein (D3YN47) may be involved in 15 metabolic pathways, including pyruvate metabolism, terpenoid biosynthesis, synthesis and degradation of ketone bodies, etc. Acetyl-CoA C-acetyltransferase helps bind two acetyl-CoA molecules to form acetoacetyl-CoA [61]. This is a key step in the production of ketone bodies, which are often involved in maintaining energy homeostasis and responding to changes in the biological environment when the main mode of energy supply to plant cells is insufficient. Tong [62] et al. demonstrated that the synthesis and degradation of ketone bodies play a role in the response to Al stress in peanut. Acet-CoA C-acetyltransferase also performs a variety of roles, such as regulating phytochromes and phytohormones [63], suggesting that this protein plays a pivotal role in carbohydrate metabolism. In addition, many upregulated DEPs were involved in amino sugar and nucleotide sugar metabolism in carbohydrate metabolism; five DEPs in GNR2 and six DEPs in GNR4 were involved in this metabolic pathway. Amino sugar and ribonucleotide metabolism performs multiple important roles in plants, involving cell wall synthesis, glycoprotein synthesis, energy storage and transfer, and key biological processes, such as growth and developmental regulation [64,65]. Therefore, amino sugar and nucleotide sugar metabolism may be an important defense mechanism in *C. semiserrata* under aluminum stress.

Interestingly, the expression of Ribulose bisphosphate carboxylase large chain (T1WY53), a component of Rubisco, was decreased under GNR4, and Rubisco catalyzes the fixation of carbon dioxide and is the only enzyme in photosynthesis that can convert atmospheric CO_2_ into organic molecules [66]; a decrease in its expression directly affects the P*_n_*, resulting in a decrease in the P*_n_*. Then, due to the decrease in carbon fixation, chlorophyll in the chloroplasts may be subjected to oxidative damage, which may cause the leaves to turn yellow. This is consistent with the results of photosynthesis under GNR4 and may cause the decrease in P*_n_* due to sufficient intercellular CO_2_ concentration under GNR4. In addition, the experimental results of Dai et al. showed a positive correlation between the activity of Rubisco and the synthesis and metabolism of total nonstructural carbohydrates (TNCs) [60]. Nonstructural carbohydrates (NSCs), including soluble sugars and starch, are important reactants for plant life activities and metabolic processes [67]. The downregulation of the expression of ribulose bisphosphate carboxylase large chain under the condition of GNR4 in this experiment may lead to a decrease in the content of cellular NSCs, and the decrease in the content of NSCs indirectly leads to a decrease in the content of SS, which is consistent with the results of physiological experiments. In addition, two other DEPs were involved in the pentose phosphate pathway (PPP), PfkB domain-containing protein (A0A4S4F1X7) and Transaldolase (A0A7J7HQU6), and the expression of these DEPs was downregulated. PPP is an important component of carbohydrate metabolism and originates from the glucose metabolic pathway. A portion of glucose molecules passes through the PPP rather than directly into the glycolytic pathway, while the PPP generates NADPH, which is a very important reducing coenzyme. NADPH plays a key role in lipid synthesis, nitrate reduction, antioxidant reactions, and other reductive metabolic processes. Ofoe treated tomato (*Solanum lycopersicum* L., ‘Scotia’) seedlings with different concentrations of aluminum (0, 1, and 4 mmol/L) and found that Calvin–Benson cycle (CBC) and pentose phosphate pathway (PPP) metabolites were significantly reduced under 4 mmol/L stress [68]. Combined with the above data, the results indicate that a series of positive carbon hydride metabolism processes were performed in the leaves of *C. semiserrata* under aluminum stress; however, with the increase in aluminum stress concentration, the downregulated DEPs of this metabolic process in GNR4 were much larger than those in GNR2, indicating that carbohydrate metabolism was inhibited. In addition, these downregulated DEPs were involved in energy synthesis processes, leading to the inhibition of energy supply processes.

## 5. Conclusions

In this study, we comprehensively compared and analyzed the physiological and proteomic changes in *C. semiserrata* at Al stress concentrations of 1, 2, 3, and 4 mmol/L with the control. The results showed that under lower concentrations of Al stress, *C. semiserrata* resisted Al stress-induced damage by enhancing the activity of the antioxidant enzyme system and increasing the content of osmoregulatory substances. At this time, the rate of cellular scavenging of ROS was greater than the rate of ROS production by stress, photosynthesis was enhanced under this condition, and the plant did not show symptoms of excessive Al toxicity. When the concentration increased to 4 mmol/L, the accumulation rate of ROS in the leaf cells far exceeded the scavenging ability, resulting in a significant increase in the degree of membrane lipid peroxidation, which affected normal physiological metabolism. The expression levels of DEPs identified using 4D-label-free technology were generally increased for DEPs involved in photosynthesis, flavonoid metabolism, antioxidant and defense, and carbohydrate metabolism, whereas the level was generally decreased for those involved in energy metabolism. In conclusion, through analyzing the physiological responses and proteome of *C. semiserrata*, we identified the key pathways and proteins involved in Al stress, providing a basis for further studies on its response mechanism to Al stress and laying a solid theoretical foundation for the development of oil tea resistance to stress.

## Figures and Tables

**Table 1:** Basic protein identification information.

Database	Peptides	Unique Peptides	Protein Groups
UniProt taxonomy	26,027	14,767	3960

Notes: Database: the name of the database species used; Peptides: total number of different peptides identified; Unique peptides: total number of unique peptides identified; Protein groups: total number of proteins identified.

**Table 2:** A portion of key proteins in the leaves of *C. semiserrata* seedlings under aluminum stress. “–” indicates that the protein did not meet the screening conditions of fold change ≥ 1.2 or fold change ≤ 1/1.2 with *p* value < 0.05 or was not detected at this stage.

Accession	Gene Name	*p*-Value		FC		Protein Name
		GNTR2/GNR0	GNTR4/GNR0	GNTR2/GNR0	GNTR4/GNR0	
Photosynthesis-related proteins						
A0A067YPP4	psbD	1.68 × 10^−2^	–	Down	–	Photosystem II D2 protein
A0A7J7GLK3	HYC85_019355	3.00 × 10^−5^	3.00 × 10^−5^	Down	Down	Divinyl chlorophyllide a 8-vinyl-reductase, chloroplastic
A0A7J7I432	HYC85_000596	4.96 × 10^−2^	–	Down	–	Thioredoxin domain-containing protein
A0A4V3WJ29	TEA_026548	4.00 × 10^−2^	–	Down	–	CP12 domain-containing protein
A0A7J7H8S9	HYC85_014962	7.00 × 10^−5^	–	Up	–	Glyceraldehyde-3-phosphate dehydrogenase
A0A4S4EUG2	TEA_005193	3.00 × 10^−5^	–	Up	–	Aspartate aminotransferase
A0A4S4F2H6	TEA_019916	–	5.30 × 10^−4^	–	Up	Photosystem I reaction center subunit VI
A0A7J7GPC2	HYC85_019403	–	3.00 × 10^−5^	–	Down	Ferredoxin
Flavonoid biosynthesis-related proteins						
H9AZQ2	CHS3	1.00 × 10^−5^	6.00 × 10^−5^	Up	Up	Chalcone synthase 3
Q6DV45	F3H	1.20 × 10^−4^	1.17 × 10-3	Up	Up	Flavanone 3-hydroxylase
A0A6N0C6H2	DFR	2.00 × 10^−5^	9.90 × 10^−4^	Up	Up	Dihydroflavonol 4-reductase
A0A4S4DFF3	TEA_023636	1.00 × 10^−4^	–	Up	–	Amino acid transporter transmembrane domain-containing protein
A0A481Y6T5	ANR2	–	2.95 × 10^−2^	–	Up	Anthocyanidin reductase
Antioxidant-related proteins						
A0A4V3WMM0	TEA_004243	2.58 × 10^−2^	–	Down	–	Superoxide dismutase
A0A4S4EEB5	TEA_015634	9.00 × 10^−4^	–	Up	–	Peroxidase (*C. sinensis* var. sinensis)
A0A858CAD6	POD5	–	3.70 × 10^−2^	–	Down	Peroxidase 5 (*C. sinensis*, Tea)
W6ACL7	HYC85_011867	–	2.59 × 10^−2^	–	Down	Glutathione transferase (*Camellia japonica*)
A0A7J7HB73	HYC85_011868	–	4.20 × 10^−4^	–	Up	GST N-terminal domain-containing protein
A0A7J7HD86	HYC85_011877	–	1.00 × 10^−4^	–	Up	GST C-terminal domain-containing protein
Carbohydrate metabolism-related proteins						
A0A7J7HJQ1	HYC85_010601	2.59 × 10^−3^	–	Down	–	ECH_2 domain-containing protein
A0A4S4DF70	TEA_028203	7.00 × 10^−5^	–	Up	–	3-hydroxyisobutyryl-CoA hydrolase
M9RZ20	GME	3.76 × 10^−2^	1.43 × 10^−2^	Up	Up	GDP-mannose-3’,5’-epimerase
D3YN47	AACT	1.65 × 10^−2^	2.02 × 10^−2^	Up	Up	Acetyl-CoA C-acetyltransferase protein
A0A4S4EMY3	TEA_006816	5.10 × 10^−4^	1.00 × 10^−5^	Up	Up	Glycine cleavage system H protein
T1WY53	rbcL	–	2.06 × 10^−2^	–	Down	Ribulose bisphosphate carboxylase large chain
A0A4S4F1X7	TEA_020366	–	4.06 × 10^−2^	–	Down	PfkB domain-containing protein
A0A7J7GNN9	HYC85_019163	–	3.28 × 10^−3^	–	Down	Dihydrolipoyl dehydrogenase
A0A7J7HQU6	HYC85_007230	–	1.60 × 10^−2^	–	Down	Transaldolase

## Data Availability

Data are contained within the article and Supplementary Materials.

## Supplementary Materials

The following supporting information can be downloaded at: https://www.mdpi.com/article/10.3390/genes15010055/s1, Table S1: Measurement data of photosynthesis parameters; Table S2: Differentially expressed proteins qualitative and quantitative results; Table S3: Differential expression proteins screening results.
